# Impact of a personalised, digital, HIV self-testing app-based program on linkages and new infections in the township populations of South Africa

**DOI:** 10.1136/bmjgh-2021-006032

**Published:** 2021-09-02

**Authors:** Nitika Pai, Aliasgar Esmail, Paramita Saha Chaudhuri, Suzette Oelofse, Marietjie Pretorius, Gayatri Marathe, Jana Daher, Megan Smallwood, Nicolaos Karatzas, Mohammed Fadul, Anna de Waal, Nora Engel, Alice Anne Zwerling, Keertan Dheda

**Affiliations:** 1 Centre for Lung Infection and Immunity, Division of Pulmonology, Department of Medicine and UCT Lung Institute, University of Cape Town, Cape Town, South Africa; 2 South African MRC Centre for the Study of Antimicrobial Resistance, University of Cape Town, Cape Town, South Africa; 3 Faculty of Infectious and Tropical Diseases, Department of Infection Biology, London School of Hygiene and Tropical Medicine, London, UK; 4 Department of Epidemiology, Biostatistics & Occupational Health, McGill University, Montreal, Québec, Canada; 5 Department of Experimental Medicine, McGill University, Montreal, Québec, Canada; 6 Department of Health, Ethics & Society/CAPHRI, Faculty of Health Medicine and Life Sciences, Maastricht University, Maastricht, The Netherlands; 7 School of Epidemiology & Public Health, University of Ottawa, Ottawa, Ontario, Canada

**Keywords:** HIV, diagnostics and tools, screening

## Abstract

**Introduction:**

Implementation data for digital unsupervised HIV self-testing (HIVST) are sparse. We evaluated the impact of an app-based, personalised, oral HIVST program offered by healthcare workers in Western Cape, South Africa.

**Methods:**

In a quasirandomised study (n=3095), we recruited consenting adults with undiagnosed HIV infection from township clinics. To the HIVST arm participants (n=1535), we offered a choice of an offsite (home, office or kiosk based), unsupervised digital HIVST program (n=962), or an onsite, clinic-based, supervised digital HIVST program (n=573) with 24/7 linkages services.

With propensity score analyses, we compared outcomes (ie, linkages, new HIV infections and test referrals) with conventional HIV testing (ConvHT) arm participants (n=1560), recruited randomly from geographically separated clinics.

**Results:**

In both arms, participants were young (HIVST vs ConvHT) (mean age: 28.2 years vs 29.2 years), female (65.0% vs 76.0%) and had monthly income <3000 rand (80.8% vs 75%).

Participants chose unsupervised HIVST (62.7%) versus supervised HIVST and reported multiple sex partners (10.88% vs 8.7%), exposure to sex workers (1.4% vs 0.2%) and fewer comorbidities (0.9% vs 1.9%). Almost all HIVST participants were linked (unsupervised HIVST (99.7%), supervised HIVST (99.8%) vs ConvHT (98.5%)) (adj RR 1.012; 95% CI 1.005 to 1.018) with new HIV infections: overall HIVST (9%); supervised HIVST (10.9%) and unsupervised HIVST (7.6%) versus ConvHT (6.79%) (adj RR 1.305; 95% CI 1.023 to 1.665); test referrals: 16.7% HIVST versus 3.1% ConvHT (adj RR 5.435; 95% CI 4.024 to 7.340).

**Conclusions:**

Our flexible, personalised, app-based HIVST program, offered by healthcare workers, successfully linked almost all HIV self-testers, detected new infections and increased referrals to self-test. Data are relevant for digital HIVST initiatives worldwide.

**Key questions**

**What is already known?**

- The WHO has called for evidence on data for digital supports and use of community-based healthcare workers to improve services associated with HIV self-testing (HIVST).
- Data for HIVST with digital supports from Southern Africa, especially with app-based programs, remain sparse.

**What are the new findings?**

- Our healthcare workers offered a flexible, personalised program, with choice of venue and strategy, customised to preferences of participants.
- Participants who showed up to test in clinics were recruited. We also documented the standard of care in the neighbouring clinics.
- Over an 18-month period, we linked all positive and negative unsupervised HIVST and supervised HIVST participants to antiretroviral treatment initiation and preventative care pathways. We detected new infections and referrals to self-test with the program.

**What do the new findings imply?**

- This quasirandomised transition-to-scale real-life study that mimicked a real-life implementation of HIVST with digital supports.
- Findings imply a possibility to link participants to care, with a trained corpus of healthcare workers, and a 24/7 linkage service to counselling, prevention and treatment initiation through a personalised, anonymized, secure, app-based digital program.
- Findings are relevant for global stakeholders who wish to deploy such programs to young, digitally savvy populations in the region. It offers data to guide scale up of such strategies for HIV, related coinfections and COVID-19.

## Introduction

### Background and rationale

In 2016, the WHO recommended HIV self-testing (HIVST) for individuals living with undiagnosed HIV infection to know their serostatus.^1^ Performed with an approved self-test, HIVST offered preliminary screening test results, and laboratory-based confirmatory testing was deemed essential.^1–4^ Research evidence on HIVST has accumulated exponentially^5–7^ with reported increases in self-test uptake,^8–10^ detection of new HIV infections, increases in test frequency and partner referrals.^11–13^

Recently, the WHO has called for evidence in HIVST in the following areas: (A) digital innovations, (B) community health workers and peer counsellors for operationalising linkages and (C) social network use for key populations.^14^ Global data on scalable HIVST service delivery models with digital support tools that work in real-life settings remain sparse.^15–17^

With digital support tools, linkages to antiretroviral treatment (ART) and prevention services (ie, pre-exposure prophylaxis, partner notification and medical circumcision), tracking and surveillance are possible.^18–20^ With COVID-19 induced lockdowns and restrictions faced globally, the demand for digital tools has grown exponentially in countries.^18–20^ With an increasing availability of 4G/5G networks, together with an increase in ownership of smartphones/tablets in low and middle-income countries, there is an opportunity to scale digital solutions.

Global foundations and initiatives are looking for evidence to scale HIVST strategies/programs with digital support tools; yet for South Africa, data and evidence remain sparse. Furthermore, the impact of preferred choice of HIVST strategy, venue, and customized linkages, offered by health care workers and peer counsellors has never been explored.

To address these evidence gaps, we report an evaluation of a choice-based, personalised digital app/platform-based HIVST program, offered by healthcare workers and peer counsellors, in the township communities of Cape Town, South Africa.

### Objectives

We set out to compare an offer of our oral HIVST program with digital supports, offered as supervised or unsupervised HIVST, to conventional HIV testing (ConvHT) (conventional rapid and lab tests+onsite counsellor) on impact outcomes (ie, linkages, new infections and referrals to test).

### Hypotheses

We conservatively hypothesised that the proportion of participants (self-testers) linked to post-test counselling and care (ie, ART initiation) and related prevention services (ie, medical circumcision, pre-exposure prophylaxis and partner notification) and the proportion of new infections detected in self testers will be comparable in both ConvHT and HIVST arms, whereas the proportion of self-test referrals (within/to social networks) in the HIVST arm would be higher (twofold, due to its ability to reach testers) than the ConvHT arm.

## Methods

### Eligibility criteria

Included participants were 18 years or older, of unknown HIV status at baseline (past 3 months), with access to an Android/iPhone smartphone or ability to use a tablet/smartphone for HIVST. Excluded participants were either on antiretroviral therapy (ART), or with a confirmed HIV diagnosis, or a serious medical condition requiring hospitalisation.

### Study design, recruitment setting and location

The evaluation was conducted in Cape Town, South Africa, between January 2017 and June 2018. A community clinic-based quasirandomised trial study design was used to generate real-life field implementation data with an intention to scale the intervention on completion.

Convenience sampling was used to recruit participants. Members of the township population presenting to test for HIV by self-referral or referral by self-testers were recruited.

### Patient and public involvement

Participants or the public were not involved in the design, or conduct, or reporting, or dissemination plans of our research.

### Recruitment/sampling

We created a sampling frame of all clinics served by the University of Cape Town. Before study initiation, we geo-mapped all the districts in Western Cape and generated a random number sequence in STATA V.12. Within each district, from the sampling frame, we randomly sampled two geographically separated clinics to offer either HIVST or ConvHT for a total of six clinics in all.

Our study recruitment period was from January 2017 to June 2018.

We recruited individuals who presented for HIV testing to the community outreach clinics and those who met the eligibility criteria. Study participants were encouraged to refer self-testing to their partners, friends and family within their close social networks. Individuals referred by self-testers that participated in the study were also recruited. Our clinic staff recruited participants during routine and drop-in visits.

For study promotions, community outreach by healthcare workers, word of mouth, handouts/flyers, demonstration videos in clinics, a Facebook page and radio/TV announcements were deployed.

### Designing and developing the innovation

**Figure 1:**
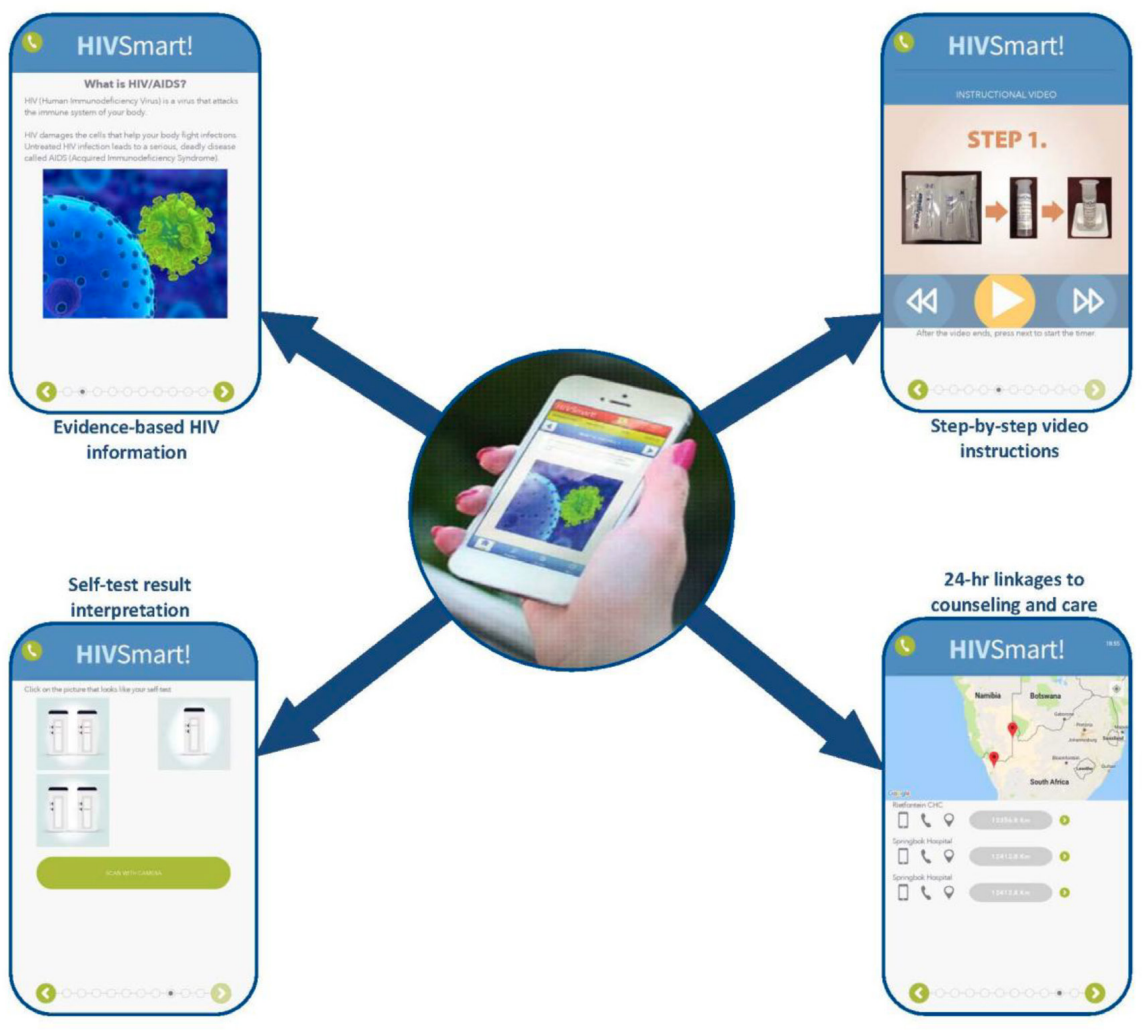
The HIVSmart! app-based digital program of care operated by healthcare workers and peer counsellors in Cape Town, South Africa.

In 2009, in Canada, we first developed a web-based HIVST strategy using design thinking principles and evaluated a prototype for usability (HIVSmart!, McGill University, 2013).^21^ Subsequently, we evaluated the strategy for feasibility in South Africa^22^ and Canada.^23 24^ Funded by the Governments of Canada and South Africa, we expanded our strategy to a digital HIVST app-based program of care in 2015 (figure 1). We added the following features for personalisation: multilingual content in five languages (ie, Xhosa, Afrikaans, Zulu, English and French); a device agnostic, Android/iOS portable platform (ie, tablet, web, smart/mobile phone); added simplified video instructions for HIVST; self-test interpretation/capture with data storage; and a 24/7 preferred clinic based service that offered pretest/post-test counselling/linkage service to treatment and prevention pathways, in their preferred language, to nearby clinics. The counselling and linkage service was offered 24/7 by trained healthcare workers and peer counsellors (ie, over phone calls, texts, chats, messages and face to face). We housed our program on a secure Health Insurance Portability and Accountability Act (HIPAA)-compliant cloud server and added a user-friendly dashboard and a global positioning system clinic locator, which was useful to both patients and providers.^22 23^

#### HIVST arm

Our intervention consisted of our innovative digital HIVST program together with an oral HIV self-test (OraQuick advance HIV-1/2, OraSure Technologies Inc, USA). Our mobile app was downloaded onto the participant’s phone or tablet (using QR codes). Participants were provided a Wi-Fi access card for connectivity.

From each participant, the research nurse obtained informed consent, explained the study procedure, performed standard per clinic protocols (rapid tests and reference standard testing for the labs) to provide consistent reference standard data and collected data on sociodemographic and test experience.

#### Delivery of the intervention

We offered a choice of two strategies and associated venues to self-test, together with customised linkages to preferred clinics for ART initiation and follow-up chosen by participants. No financial incentives and no phones were offered to study participants. Only Wi-Fi access cards for connectivity were provided to make it easy for the participants to communicate with the healthcare workers through the app via text messages/calls. The phones belonged to the participants. Research staff who did not own phones were provided with smartphones and a Wi-Fi access card for the entire duration of the study for communication purposes only.

The procedures for each HIVST option to test were as follows:

1. Supervised digital HIVST at the clinic: participants performed HIVST in the presence of a counsellor or healthcare worker. For this option, participants were provided a tablet with the downloaded app, an oral self-test and a Wi-Fi access card to call counsellors. Participants were ushered to a private space set up in the clinic to perform the self-test. After self-test was conducted, participants could drop their self-test in a drop box outside the clinic. Total duration of intervention was 1.5 hours (including orientation and testing). The tablet was housed in the clinic and was secured with a lock to prevent it from being taken home by anyone. Tablets were collected from the clinic at the end of the day and stored in a locked drawer in the offices of the principal investigator.
2. Unsupervised digital HIVST at a venue of their choice: the research nurse offered participants the option to conduct their self-tests unsupervised anywhere: at home, at their office or workspace or in private space (kiosk). The participants were provided with an oral self-test, a QR code and a Wi-Fi access card to call counsellors. Orientation time of 30 min, and unsupervised test program of 1 hour, for a total duration of 1.5 hours.
  The phones belonged to the participants and were not provided to the participants. Participants were asked to download the QR code in the presence of the counsellor to check if the app was working. Once the app opened up, the participants were allowed to leave. Participants who were confident of testing by themselves chose the home/office option, and participants who lacked safe space to test at home or office chose to test at the kiosk. Participants were requested to drop the test kits back at the clinic in the drop off box, or bring it with them when they came back to the counsellor for care.

To arrange for linkages to counselling, disease staging or ART initiation in test positives and prevention services in test negatives, the healthcare workers/peer counsellors recorded participant’s language preferences, preferred mode of follow-up communication (chat, SMS, phone call and face to face) and their preferred clinic location (UCT vs non-UCT). This helped the healthcare workers customise their service to the choice, preference and lifestyle of participants.

#### ConvHT arm

ConvHT testing (standard of care standard rapid and lab tests) was offered in comparator clinics with onsite counsellors; duration was 1.5–2 hours with 3–4 hours of waiting time in clinics. The research nurses explained the study procedure to consenting participants, obtained informed consent and performed ConvHT (blood-based rapid tests and sample collection for reference standard), followed by data collection for sociodemographic variables and test experience. Participants were asked to refer their partners and friends and family to test for HIV at these clinics. Linkage and counselling service followed the model used for the ConvHT conducted in two steps. The first step in linkage seeking is post-test counselling and sharing of lab confirmed test result. This is followed by blood draw for disease staging of lab confirmed test positives and risk reduction counselling and triage to prevention services (ie, medical circumcision, pre-exposure prophylaxis and partner notification) for lab confirmed test negatives. The second step was ART initiation at the clinic based on all lab results for test positives.

### Outcomes

To evaluate the impact on the primary outcome, linkages to counselling, ART initiation and prevention services, we documented comparative linkage data from the ConvHT arm.

Linkage was defined in two ways:

1. Linkage to counselling, a proportion, was computed by documenting numerator/denominator. Numerator was compiuted as the number of both self-test positive and self-test negative participants who communicated and showed up for face-to-face post-test counselling, receipt of lab-confirmed test results, followed by disease staging of lab confirmed test positives, or triage to prevention services for lab confirmed test negatives. Denominator was defined as the total number of consenting participants who sought self testing.
2. Linkages to care was defined as the proportion of newly identified participants linked to clinics for ART initiation (numerator) over total number of test positives identified in the study (denominator).

Proportion of linkages in each arm were documented, and comparisons were reported with risk ratio (RR) and associated 95% CIs.

For the HIVST arm, we recorded receipt of linkage services through the application; when a participant called for counselling, a phone call or virtual chat occurred, followed by a face-to-face meeting for services. Linkages to treatment for positives in preferred clinics were confirmed by date and corroborated with record of receipt of ART from clinic rosters by healthcare workers. Similarly, linkages to prevention services for test negatives were confirmed and corroborated with clinic records maintained by healthcare workers.

To evaluate the impact on the secondary outcome, *detection of new infections*, we compared and documented the proportion of newly identified participants as test positives in both arms. Test positives/negatives were confirmed by lab-confirmed results and test protocols. For both arms, we calculated the total number of new infections detected and staged (numerator) over the total number of individuals that sought testing (denominator). To compare new infections, we considered the test results (against a reference standard of rapid tests and lab-confirmed tests) separately, and computed RR (95% CI).

To evaluate the impact on tertiary outcome, *referrals to HIVST*, separately for each arm (HIVST and ConvHT), we computed the total number of referrals to test made by a self-tester (phone-based and word-of-mouth referrals) to someone in their social network (ie, partners, spouses, friends, family and community members). Next, for each arm, we compared the differences in estimates from the end of the study to baseline. Referrals were documented by our research nurses who explained the choices of strategy or venues to test and inquired about their reasons and motivations for referrals. Referrals were not linked to primary self-tester to avoid a breach of confidentiality. Proportions were compared, and RRs (95% CI) were computed.

To document choice, we computed the proportions by calculating the total number of consenting HIVST participants (denominator). We calculated HIVST participants who chose either strategy, supervised HIVST(one venue: clinic based), versus, unsupervised HIVST (three venues: home, office/prviate space and kiosk) for the numerator.

### Sample size

Assuming an equivalence margin of 10%, success proportion for linkage of 85% for HIVST arm and 90% for the conventional testing arm (alpha 5%; power of 95%), a sample size of 2262 participants (1131 in each arm) was deemed sufficient for our linkage estimations. Assuming an attrition rate of 10%, sample size of 1500 per cohort was estimated to be adequate to detect comparable linkages and new infections between HIVST and ConvHT and to detect a conservative twofold increase in referral in the HIVST cohort compared with ConvHT cohort, with a CI±10%.

### Assignment method

The unit of assignment was the individual participant. Although our participants, investigators and staff were open (unblinded) to the intervention, outcome assessors (statisticians) were blinded to the intervention assignment. Assignments were revealed to them at the end of their analyses. Assignment was restricted to clinics served by University of Cape Town.

### Data sources, collection and measurement

For deidentification of each participant, a unique study ID number was created. It identified the site, the township clinic and the program (HIVST or ConvHT). Likewise, each self-test referral was coded by a reference number that identified the site, the township clinic and the program, self-reported by the study participant and recorded.

For both arms, we collected deidentified, encrypted, digital data on the app linked reference standard laboratory test data. For the ConvHT arm, baseline characteristics (table 1) data were collected using digital case report forms with anonymised data on exposure, outcome and confounders. Laboratory data collected on tablets were supplemented by data from HIV registers/folders.

**Table 1:** Baseline characteristics of participants

Characteristics	Self-testing arm (n=1535)	Conventional testing arm (n=1560)	Difference (95% CI)
**Age, mean (SD**)	28.23 (8.83)	29.18 (8.56)	− 0.95 (−1.56 to 0.34)
NA=missing (n (%))	0 (0.00)	1 (0.06)	−0.0006 (−0.0018 to 0.0006)
**Gender** (**n (%) male**)	541 (35.24)	378 (24.23)	0.11 (0.08 to 0.14) *
NA=missing	0 (0.00)	1 (0.06)	−0.0006 (−0.0018 to 0.0006)
**Education (n (%**))			
0=no schooling OR primary school	114 (7.43)	100 (6.41)	0.01 (−0.01 to 0.03)
1=high school OR more advanced	1420 (92.51)	1460 (93.59)	−0.01 (−0.03 to 0.01)
NA=missing	1 (0.07 %)	0 (0.00 %)	0.0007 (−0.0006 to 0.0020)
**Work situations (n (%**))			
0=employed (full time)	337 (21.95)	567 (36.35)	−0.14 (−0.18 to 0.11)*
1=employed (part time)	207 (13.49)	177 (11.35)	0.02 (−0.002 to 0.044)
2=not employed	916 (59.67)	812 (52.05)	0.07 (0.04 to 0.11)*
3=retired	12 (0.78)	4 (0.26)	0.005 (0.0001 to 0.0103)*
NA=missing	63 (4.10)	0 (0.00)	0.041 (0.031 to 0.051)*
**Monthly income (n (%**))			
0 =<3000 rand	1190 (77.52 %)	1167 (74.81)	0.03 (−0.002 to 0.06)
1=3000–6000 rand	153 (9.97)	304 (19.49)	−0.09 (−0.12 to 0.07)*
2=6000–9000 rand	61 (3.97)	46 (2.95)	0.01 (−0.003 to 0.023)
3 =>9000rRand	68 (4.43)	40 (2.56)	0.02 (0.01 to 0.03)*
NA=missing	63 (4.10)	3 (0.19)	0.04 (0.03 to 0.05)*
**Source of income (n (%**))			
0=employed	531 (34.59)	742 (47.52)	−0.13 (−0.16 to 0.09)*
1=family or friends	947 (61.69)	766 (49.10)	0.13 (0.09 to 0.16)*
2=grant	47 (3.06)	46 (2.95)	0.001 (−0.01 to 0.01)
3=pension	9 (0.59)	6 (0.38)	0.002 (−0.003 to 0.006)
NA=missing	1 (0.07)	0 (0.00)	0.007 (−0.0006 to 0.0020)
**Previous HIV test (n (%**))			
0=first-ever HIV test	75 (4.89)	58 (3.72)	0.01 (−0.002 to 0.026)
1=repeat HIV test (last test less than 1 year ago)	915 (59.61)	1021 (65.45)	−0.06 (−0.09 to 0.02)*
2=repeat HIV test (last test more than 1 year ago)	544 (35.44)	481 (30.83)	0.05 (0.01 to 0.08)*
NA=missing	1 (0.07)	0 (0.00)	0.0007 (−0.0006 to 0.0020)
**Current partner your husband or wife?** (**n (%) yes**)	429 (27.95)	432 (27.69)	0.002 (−0.03 to 0.03)
NA=missing	69 (4.50)	12 (0.77)	0.04 (0.03 to 0.05)*
**Are you sexually active? (n (%) yes**)	1223 (79.67)	1481 (94.94)	−0.15 (−0.17 to 0.13)*
NA=missing	74 (4.82)	0 (0.00)	0.05 (0.04 to 0.06)*
**In the past 6 months, sex with multiple partners (n (%) yes**)	167 (10.88)	136 (8.72	0.02 (0.001 to 0.042)*
NA=missing	81 (5.28)	0 (0.00)	0.05 (0.04 to 0.06)*
**In the past 6 months, sex with a sex worker (n (%) yes**)	21 (1.37)	3 (0.19)	0.01 (0.005 to 0.018)*
NA=missing (%)	81 (5.28)	0 (0.00)	0.05 (0.04 to 0.06)
**In the past 6 months, sex with an HIV-infected partner (n (%) yes**)	42 (2.74)	35 (2.24)	0.005 (−0.006 to 0.015)
NA=missing	81 (5.28)	0 (0.00)	0.05 (0.04 to 0.06)
**Have you ever been diagnosed with:**			
Tuberculosis (n (%) yes) *	121 (7.88)	126 (8.08)	−0.002 (−0.02 to 0.02)
Other lung infection (n (%) yes) *	6 (0.39)	5 (0.32)	0.0007 (−0.003 to 0.005)
Diabetes (n (%) yes) *	14 (0.91)	29 (1.86)	−0.009 (−0.0177 to 0.0012)*
Hypertension (n (%) yes) *	46 (3.00)	128 (8.21)	−0.05 (−0.07 to 0.04)*
Asthma (n (%) yes) *	36 (2.35)	81 (5.19)	−0.04 (−0.05 to 0.02)*

*No missing data in both arms.

For the HIVST arm, anonymised data on exposure, outcome, confounders, laboratory data, review of HIV registers, encrypted data on the app and the server were similarly collected. Access to the HIPAA-compliant platform was granted only to the two principal investigators involved in the study. Each day, data from the app were corroborated with lab data and clinic data. Access to recruiting staff was restricted to data entry only.

A regular oversight and monitoring of recruitment was possible with a dashboard. Daily record of numbers improved data quality and provided a snapshot to the key personnel involved in the study. Data on ART clinic initiation and lab results were corroborated with clinic rosters. Some participants preferred to link to clinics outside the University of Cape Town (UCT) system. To allow for this, permission was sought from the city to collect data from non-UCT clinics by study staff. All self-tests were confirmed by rapid tests and laboratory tests as per clinic protocols.

### Statistical methods

A descriptive analysis on characteristics of all study participants was performed. For the continuous covariates, we reported mean (SD), and for categorical covariates, we reported % in each category. We replaced missing covariate values by the most frequent category (categorical variables) or the median (continuous variables).

We considered outcome metrics (ie, linkages, new infections and referrals) as binary and defined success and failure appropriately. For each outcome, we compared the proportion of success between the HIVST and ConvHT arms. We performed the statistical z-test to compare two proportions, and we reported the RR and 95% CI for our outcomes.

An RR >1 indicated that the HIVST arms were favourable (linkage, new infections and higher referral), while an RR <1 indicated that the ConvHT arm was favourable.

To control for confounding at baseline (to reduce selection bias), we performed a propensity-matched analysis of outcomes. Variables for the propensity model (ie, age, gender, Socio-economic status (SES), work status, sexual history and comorbidities) were decided a priori based on their significance and their impact on participant’s choice and preference. A nearest-neighbour matching criterion was used to identify comparable subjects in each cohort. Following the identification of the matched cohort, we estimated the RR and CI for each of the outcomes as mentioned by Austin^25^ and performed our Statistical analyses in R (V.3.3.0).

## Results

### Demographics and flow

**Figure 2:**
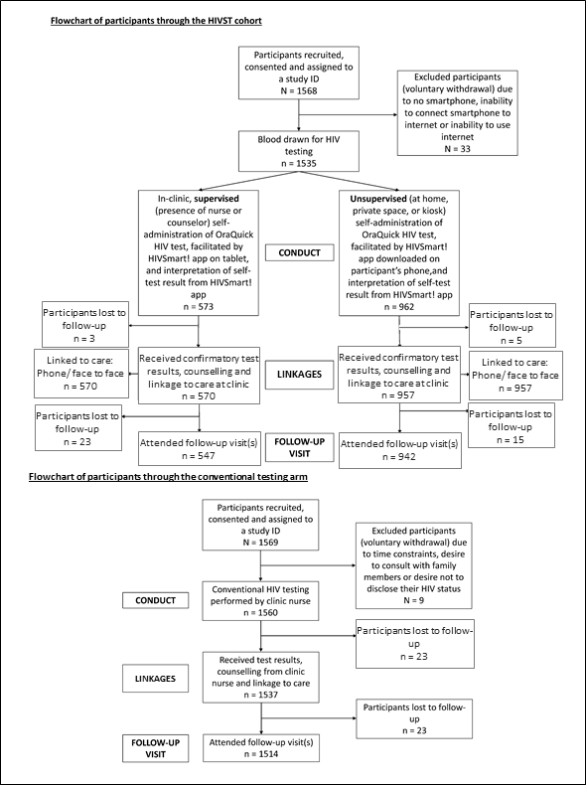
Flow diagram of participants through the HIVST and ConvHT arms. ConvHT, conventional HIV testing; HIVST, HIV self-testing.

A total of 3137 participants were enrolled, of which 33 in the self-testing arm and nine in the conventional arm opted out (reasons are documented in figure 2).

Of the 3095 participants, 1535 participants in the HIVST arm and 1560 participants in the ConvHT were recruited, respectively. A majority of participants in the HIVST arm (n=962, 62.7%) chose unsupervised HIVST over supervised HIVST (n=573, 37.3%).

Sociodemographic data on participants in HIVST versus ConvHT arm demonstrates that participants in both arms were young (mean age: 28.2 years vs 29.2 years), female (65.0% vs 76.0%) and poor <3000 rand (US$253) (80.8% vs 75.0%).

Participants in the HIVST arm versus ConvHT arm reported significant differences in the following variables: sex with multiple partners (11.5% vs 8.7%), exposure to sex workers (1.4% vs 0.2%), less sexual actiivty (79.6% vs 94.9%), with repeat HIV test more than a year (35.4% vs 30.8%), repeat HIV test less than a year (59.6% vs 64.5%) and HIVST participants versus ConvHT arm participants were also relatively healthier with fewer comorbidities (diabetes: 0.9% vs 1.9%; hypertension: 2.9% vs 8.2% and asthma: 2.35% vs 5.19%). More men attempted to know their status through HIVST versus ConvHT (35.24% vs 24. 23%).

Please refer to table 1 for baseline sociodemographic characteristics.

### Outcomes

Compared with ConvHT (98.5%), in the HIVST arm, our primary linkage outcome to counselling and care was high at 99.7% (break up: unsupervised HIVST arm: 99.8%; supervised HIVST arm at 99.3%) (unadjusted RR: 1.012; 95% CI 1.005 to 1.018; propensity-adjusted RR: 1.011; 95% CI 1.005 to 1.018). Our secondary linkage outcome for ART initiation for HIV test positives and prevention pathways for HIV negatives was ((overall digital HIVST: 98.1%; supervised HIVST: 95.7%; unsupervised HIVST: 99.3%)) and comparable with ConvHT: 98.5%.

At 1 month, we documented the losses to follow-up in each arm. In the HIVST arm, we had 38 participants (break up: 23 supervised, 15 unsupervised arm) versus 23 participants in the ConvHT arm.

Regarding new infections, compared with ConvHT arm at 106/1560 (6.79%), we detected a slightly overall higher proportion (9%) in the HIVST arm (break up: unsupervised HIVST at 73/962 (7.6%) and supervised HIVST at 63/573 (10.9%)) (RR: 1.305; 95% CI 1.023 to 1.665). Propensity-adjusted analysis showed marginally better performance in the HIVST arm versus ConvHT (RR: 1.295; 95% CI 1.011 to 1.665).

As for referrals to self-test within their social network (ie, partners, family and friends), it was proportionally higher in the HIVST arm (16.7%) versus ConvHT arm (3.1%) (RR: 5.435; 95% CI 4.024 to 7.340). The propensity-adjusted analysis preserved the RR (adjusted RR: 5.391; 95% CI 3.992 to 7.281).

Regarding choice, an overwhelming majority (n=962 (62.67%)) chose the unsupervised HIVST strategy, while a few (n=573 (37%)) chose supervised HIVST. Participants were 1.70 times more likely to choose the unsupervised HIVST over supervised HIVST, reflecting the popularity of our digital unsupervised HIVST strategy. Among choice of venue to test unsupervised, homes and kiosks were popular followed by offices/workspaces.

Please refer to table 2A, B for analyses, respectively.

**Table 2:** Multivariate analyses and propensity score matched analyses.

A: multivariate analyses; adjusted risk ratio (RR) and risk differences with 95% CI for outcomes of interest; reference group conventional arm		
	RR	95% CI
Linkages	1.012	1.005 to 1.018
New infections	1.305	1.023 to 1.665
Referral	5.435	4.024 to 7.340

B: propensity-matched analyses; adjusted RR with 95% CI for outcomes of interest		
	Propensity adjusted	
	RR	95% CI
Linkage	1.011	1.005 to 1.018
New infections	1.295	1.011 to 1.660
Referral	5.391	3.992 to 7.281

## Discussion

To the participants, we offered a personalised, choice-based HIVST program with digital support tools, in a real-life implementation study design, and compared impact outcomes in HIVST versus ConvHT arms.

First, many participants in the HIVST arm (n=962 participants, 62.7%) chose unsupervised over supervised HIVST strategy, reflecting the need for autonomy, privacy and convenience to self-test. Compared with ConvHT (98.5%), our linkage proportions for counselling for HIVST were at (99.7% overall). Break up of linkage metrics for HIVST: unsupervised digital HIVST (99.8%), supervised digital HIVST (99.3%). Our linkage proportions for ART initiation in HIVST arm were comparable with ConvHT, reflecting the fact that our participants preferred the customised program offered by our healthcare workers.

Operationalising linkages has been a huge challenge in HIVST. Globally, linkage data for digital unsupervised HIVST with app-based programs are sparse. Linkage data for HIVST (without a digital component) varies between 51% and 81%^1^ and has been reported from many countries: Thailand,^26^ China,^27 28^ Vietnam,^29^ Singapore,^30^ the Americas (Brazil,^31^ USA^32–34^ and Canada),^23 24^ Australia^35^ and Europe (Spain).^36^ We believe that our customised, unincentivised, 24/7 digital program offered by our healthcare workers, together with a choice of strategy and venue to test, contributed to the success of our strategy.^37^ HIVST strategy was popular, but unsupervised HIVST appealed to the participants that wanted to exercise privacy, autonomy and independence to test anywhere. Although homes, offices were popular venues to self-test, those participants who did not find homes/offices to be safe/comfortable preferred kiosks. Exploration of choice of strategy and venue in the context of this study has been examined in great detail in related recently published qualitative research from this project.^38 39^

Retention in care was also maintained throughout the study period through digital communication. This was not entirely surprising, for we found similar retention in care estimates with our AideSmart! Multiplex app-based program for coinfections for pregnant women in rural India.^40^ Retention in care in participants was possible without incentives because of a personalised service available 24/7 that catered to their lifestyle and accommodated their choice and preferences that further enabled its success.

Second, although we detected a modest increase in new infections in the HIVST arm versus ConvHT arm (RR: 1.305; 95% CI 1.023 to 1.665), we detected many new HIV infections in both supervised and unsupervised HIVST arms; this finding concurs with cumulative evidence in HIVST.^1^ HIV incidence is explained by the popularity of the strategy in young populations. The HIV incidence was high in young women and their referrals. Perhaps, there is a need for targeted, digital strategies to engage these young digitally savvy populations.^41^

Third, we extended referrals to partners, friends and family within their social network, including those in need of HIV testing and those living with undiagnosed HIV infection and those that were not keen to test in conventional clinics.^42 43^ With that, we found a high proportion of referrals for HIVST. This finding is in line with WHO recommendations for HIVST. Secondary distribution of self-tests to partners has been successful in HIVST.^44^ Incidentally, our self-testers were predominantly young women, but our referrals were predominantly men. Rapid information sharing by women to men was possible through our digital HIVST app that actively engaged young men.^45^ We concur that women could be the change agents for HIVST- by encouraging men within their social network to self-test, with a digital program. they can expedite progress towards UNAIDS targets. Our finding is aligned with HIVST studies^43 46 47^ and supports current WHO recommendations.

A few elements of our HIVST program have contributed to its success. These are: (A) deployment of a complete app-based program with digital supports, (B) offering a choice of strategy, a choice of venue to self-test and a choice to link participants to their preferred clinics for ART and prevention pathways, (C) provision of customised communication in the participant’s language, together with a choice of communication modalities (ie, text, calls, chat, followed by face to face), encouraged a bidirectional exchange impacting participant engagement; (D) continued presence of our team of healthcare workers with a 24/7 service that included facilitating counselling, linkage to ART initiation at the nearest preferred clinic, facilitating follow-up and linkages to prevention services; and lastly (E) task shifting of counsellors and healthcare workers, from performing primary screening in clinics to optimising linkages.^46^

### Strengths

Our study strengths lay in its novelty, its digital program, its large sample size, representative digitally savvy young populations and flexible real-life implementation study design. A quasirandomised study design offered the flexibility to understand choice of strategy, venue to test and customisation of counselling and linkage services based on self-tester’s preference and comfort level with technology. It was not possible to explore choice nor offer flexibility, within a randomised clinical trial framework. Furthermore, many such flexible designs are now being recommended for digital initiatives.^48^ We have conducted extensive qualitative research to explore the role of choice, flexibility and preferences, and customisation in detail.^38 39^ We are also completing a cost-effectiveness analysis.^49^

### Limitations

Our data are comparable, as confirmed by negligible baseline differences (balance) in the participants’ demographics minimising sampling bias. A random selection of study and comparator sites in the design stage minimised selection bias. A complete reporting of all deidentified outcomes and blinding our statisticians to the intervention assignment minimised detection bias. Propensity-adjusted modelling minimised confounding at baseline. Propensity scores do not account for latent characteristics, which could not be ruled out. Social desirability bias could not be ruled out. Despite these limitations, our analyses showed similar outcomes, and matched results in both arms favoured a digital HIVST over ConvHT.

Inherent risks of self-testing without an app-based guided program are namely: (A) failure of self-test as described in the self-test brochures, (B) failure to record and log their self-test result accurately, (C) failure to call for counselling when in need and (D) failure to seek linkage to ART care and initiation in nearest clinics. These risks exist for an unsupported program and cannot be discounted. Although app-based programs and web programs if adequately supported and maintained can minimise these risks, but they cannot necessarily eliminate them.

### Generalisability

Our findings may be generalisable to young populations from South Africa and similar digitally savvy populations who can navigate care and counselling proactively, using an app-based program, guided by healthcare workers.

Our HIVST strategy is applicable in settings and in contexts where a committed team of counsellors is willing to work with the participants to customise their preferred choice of venue, language and linkage to clinics. Our study was restricted to Western Cape, which had a greater penetration of internet connectivity. Our recruiting clinics were served by the University of Cape Town, which has a formidable reputation in the city. The program was successful with a team of healthcare workers who were familiar with the patient populations they served and were able to navigate the healthcare systems with the study participants.

Some additional points of interest for implementers are highlighted below.

First, although we did not incentivise our program, we provided a flexible, choice-driven customised service that was greatly appreciated by participants. Flexibility and choice of venue and digital supports increased and maintained a continued engagement with our study participants. Second, this program will only work in settings where digital or Wi-Fi connectivity are uninterrupted, where participants are digitally savvy and can navigate an app-based service proactively. For those participants who had difficulty navigating their phones, we offered a choice of supervised strategy in clinics or kiosk based unsupervised HIVST strategy offered on tablets. Third, implementation challenges that were faced by us included the use of a protected HIPAA-compliant server that ran into firewall issues initially but took a month to resolve these issues. HIPAA-compliant servers are expensive but ensure data privacy and security. Fourth, some participants did not have the best Android phones (version five or higher), and they could not run the entire program on their phones due to bandwidth and storage issues, so they opted for the supervised HIVST in clinics or the kiosk based unsupervised HIVST. Fifth, mobilising ART initiation outside of UCT clinics required permission from the city. Sixth, the inherent sampling and social desirability bias in the study could impact generalisability of study findings. Those participants who desired to self-test using phones and apps consented to the study. Further exploration of these biases is needed to better customise these programs for young populations. Seventh, the continuous presence of UCT clinicians for ART initiation and care and presence of UCT trained counsellors and nurses for counselling and support for the duration of the project facilitated care. Lastly, mobilising and addressing the scepticism of some of our counsellors who were hesitant initially to embrace technology was an early challenge. The counsellors feared losing their jobs. However, as the study progressed, they engaged better with technology and found its relevance and its reach in serving populations they cared for and with speed and efficiency. After a few months, these counsellors became the champions of our program. Thus, by creating champions, the study generated clinical and public health impact data of relevance and generated social impact.

### Implications

In 2021, in the setting of COVID-19 infection, recurrent lockdowns and COVID-19 variant induced epidemics are making it difficult to offer routine screening for HIV in public settings. Now, more than ever, there is an increased demand for HIVST with digital supports and related demand for increased screening for sexually transmitted and blood-borne infections. HIVST with digital support tools can pave the way for digital self-testing/self-sampling options for related screening initiatives for hepatitis C, COVID-19, Human Papillomavirus (HPV) and Chlamydiae Trachomatis /Neisseria Gonorrheae (CT/GC).

While barriers like smartphone ownership, digital literacy, access to Wi-Fi and connectivity (digital divide) impede expanded access, they also offer opportunities to think of out-of-the-box ways to improve access. These include setting up public/private partnerships that offer a limited time ownership of smartphones or free Wi-Fi connectivity for a clinical or health service of a limited duration. Furthermore, flexible self-testing programs with digital supports should consider an affiliation with a credible organisation that offers a clinical service that is well regarded by the communities. Digital supports could be offered through flexible venues: online (through platforms), or through apps (smartphones), for homes or office use, or through kiosks in outreach clinics, malls, public and private pharmacies, or by non-government organisation-based outreach clinics, mobile vans or vending machines.^46^

Digital programs could be made available for open access for low-income countries and available for scale up in fast-track cities, through government support and smart public private partnerships.^22 50^ Funded by the Canadian Institutes of Health Research (CIHR), in the coming years, we plan to expand our digital HIVST strategy for self-testing in key populations across Canadian provinces.^51^

## Conclusion

Our choice-based, customised, self-tester centred, flexible digital HIVST program, offered by a dedicated team of healthcare workers and counsellors, successfully linked almost all self-testers to treatment and care. It detected new infections, reported an increase in referrals to HIVST and maintained engagement of participants for the duration of the project.

HIVST with digital support tools like smart apps and web platforms offers an untapped potential to fast track our progress towards UNAIDS targets for HIV elimination in many countries. Our findings find relevance in the planning and scaling of many self-testing and similar digital health initiatives worldwide.

## Data Availability

Data are available on reasonable request. All data relevant to the study are included in the article or uploaded as supplementary information. All data relevant to the study are included in the article or uploaded as supplementary information.
